# High Pretreatment LDH Predicts Poor Prognosis in Hypopharyngeal Cancer

**DOI:** 10.3389/fonc.2021.641682

**Published:** 2021-03-11

**Authors:** Jialing Wu, Kaiyun You, Changlong Chen, Huimin Zhong, Yanhui Jiang, Huaqian Mo, Juanjuan Song, Xingsheng Qiu, Yimin Liu

**Affiliations:** Department of Radiation Oncology, Sun Yat-Sen Memorial Hospital, Sun Yat-Sen University, Guangzhou, China

**Keywords:** lactate dehydrogenase (LDH), hypopharyngeal cancer, prognosis, retrospective, T3–4

## Abstract

**Background:**

Elevated pretreatment lactate dehydrogenase (LDH) has been associated with poor prognosis in various malignancies; however, its prognostic role in hypopharyngeal cancer remains elusive. In this study, we aimed to assess the association between pretreatment LDH and clinical outcome of hypopharyngeal cancer.

**Methods:**

We retrospectively collected 198 hypopharyngeal cancer patients treated with surgery in our institution between 2004 and 2018. The prognostic role of pretreatment LDH was explored by using univariate and multivariate analyses. Besides, subgroup analysis was performed based on T stage.

**Results:**

Three-year and Five-year of disease-free survival (DFS, 67.0 *vs*. 57.4%, 65.8 *vs*. 39.8%, p = 0.007) and overall survival (OS, 74.8 *vs*. 68.9%, 66.8 *vs*. 50.8%, p = 0.006) exhibited significant differences between low LDH level and high LDH level groups. Univariate analysis showed that pretreatment elevated serum LDH served as an unfavorable determinant with regard to DFS and OS. Further multivariate analysis also confirmed that LDH was an independent predictor for DFS and OS. Additionally, N status and age were also found to be significantly associated with both DFS and OS.

**Conclusion:**

Pretreatment elevated serum LDH is an inferior prognostic factor for patients with hypopharyngeal cancer. These results should be validated by more multicenter and prospective studies.

## Introduction

Among head and neck squamous cell carcinomas, hypopharyngeal cancer is comparatively uncommon and aggressive (1). Hypopharyngeal cancer occurs predominantly in males (2) with heavy alcohol consumption and tobacco use (3). It is mainly arising from pyriform sinus, and early symptoms of this malignancy are often neglected, which contributes to the fact that hypopharyngeal cancer patients usually present with an advanced stage at their first diagnosis (4, 5). Therefore, despite advancements in diagnosis and treatment modalities, the 5-year disease-free survival (DFS) rate and overall survival (OS) rate of hypopharyngeal cancer remain unsatisfied, which were in the range of 35.8–65.5% and 26.9–58.5%, respectively, according to different studies (6–14).

Surgery is one of the main initial treatment option. The surgery modality for locally advanced hypopharyngeal cancer is pharyngectomy with total or partial laryngectomy, in conjunction with selective or radical neck dissection, with reasonable adjuvant treatment based on the presence of clinical adverse pathologic features (15). Given the poor prognosis of advanced hypopharyngeal cancer, a novel prognostic determinant that can accurately predict clinical outcomes for these patients is urgently needed.

Lactate dehydrogenase(LDH) is a glycolytic enzyme (16), comprised of four polypeptide subunits, in charge of the transformation of pyruvate into lactate in aerobic conditions (17). Distinguish from non-proliferating cells, which are dominated by mitochondrial tricarboxylic acid (TCA) cycle to generate ATP needed for cellular activities, a majority of cancer cells are alternatively characterized by aerobic glycolysis, a phenomenon called “the Warburg effect.” To better understand Warburg effect, we should note that proliferating cells have more crucial metabolic requirements beyond ATP, such as carbon and NADPH, which are needed for macromolecular synthesis like nucleotides, amino acids, and lipids. Despite the inefficient ATP production of aerobic glycolysis, the superiority it conferred to cancer cells is the robust generation of NADPH and excretion of excess carbon. The enzyme LDH is the main player in the conversion of both glucose and glutamine to lactate, and one by-product of the conversion is the generation of NADPH (18). The inhibition of LDH resulted in reduced cell proliferation (19). Former research studies showed that LDH not merely associates with the activation of some proto-oncogenes, such as Myc and HIF-*α* (20), but also plays a crucial role in the maintenance of tumor invasiveness (21, 22), metastatic potential (23, 24), chemoresistance (25), and radioresistance (26). Moreover, it also showed that LDH takes part in immunosuppression (27), and LDH can be regarded as an immune surveillance prognostic marker (28) and its abnormal rise was forerunner of unfavorable outcomes in cancer patients (29).

The prognostic significance of pretreatment serum LDH in multiple malignancies is well established, such as biliary tract cancer (30), lung cancer (31, 32), breast cancer (33), gastric cancer (34), colorectal cancer (35), and so on (36). However, the clinical significance of LDH in hypopharyngeal cancer is quite unclear, and it is worthwhile to explore the prognostic role of LDH. Serum LDH is promising to become a cost effective, non-invasive, and easily reproducible marker, which can predict survival outcomes in hypopharyngeal cancer. Therefore, it is imperative to evaluate whether LDH can serve as a prognostic factor in hypopharyngeal cancer patients.

## Materials and Methods

### Patients

We retrospectively reviewed patients diagnosed with hypopharyngeal cancer between March 2004 and December 2018 in our institution (Sun Yat-Sen Memorial hospital). Exclusion criteria included: initial definitive chemoradiotherapy, no available pretreatment serum LDH, inadequate follow-up, data and renunciation of medical treatment. We enrolled the patients treated with surgery and with eligible pretreatment serum LDH. Overall, we collected 198 histologically confirmed hypopharyngeal cancer patients in this study. Patients’ baseline clinical and pathological characteristics, including age, gender, tumor differentiation, surgical margin, vascular invasion, adjuvant therapy, type of surgery, adjuvant radiotherapy, chemotherapy regimen and cycles, T stage and N status were collected and analyzed. This study was performed under the review and approval of the Institutional Review Board at Sun Yat-Sen Memorial Hospital, Sun Yat-Sen University.

### Laboratory Testing of LDH

Peripheral blood samples were collected and analyzed for laboratory tests within 2 weeks before the beginning of treatment. Serum LDH levels were measured employing a Hitachi Automatic Analyzer 7600-020 (Hitachi High-Technologies, Tokyo, Japan). Normal serum LDH enzyme levels were defined to 108–252 U/L. Based on pretreatment serum LDH levels, patients were classified into high LDH group (LDH ≥178 U/L) and low LDH group (LDH <178 U/L) (37).

### Clinical Staging

All patients underwent pretreatment evaluations, including: physical examinations, hematologic and biochemical tests, chest radiography, electronic laryngoscope, esophageal barium meal examination. Contrast-enhanced magnetic resonance imaging (MRI) or computed tomography (CT) of the head and neck was performed on 64 and three patients, respectively. MRI and CT scan were aimed to evaluate the size and surrounding invasion of the tumor, as well as the status of lymph node metastasis. Abdominal ultrasonography and whole body bone scan using single-photon emission computed tomography (SPECT), which was used to detect distant metastasis, were carried out in 143 and 85 patients, respectively.

The TNM classification of all patients were staged according to the 7th edition of the American Joint Committee on Cancer (AJCC) for hypopharyngeal cancer.

### Treatment of Patients

All of the patients received surgery with or without adjuvant therapy. Treatment option was decided upon thorough evaluation and made by a multidisciplinary team, regarding treatment efficacy, function maintenance and complication. Surgery modality including excision of hypopharyngeal cancer in the absent of neck dissection, pharyngectomy with total or partial laryngectomy, in conjunction with radical, modified radical, selective or extended neck dissection. According to the guideline proposed by the American Head and Neck Society (38) for patients with hypopharyngeal cancer, (1) radical neck dissection referred to ipsilateral cervical lymphadenectomy of levels I–V, (2) modified radical neck dissection spared one or more non-lymphatic structures, (3) lymph nodes in levels II–IV were removed in selective neck dissection, (4) removal of structures not contained in radical neck dissection termed extended neck dissection. Alterations could be made depending on the sophisticated clinical circumstances.

Adjuvant treatment was carried out based on the presence of clinical adverse pathologic features such as extranodal extension, positive margin, pT3 or pT4 primary, multiple positive lymph nodes, perineural invasion and lymphatic invasion. 119 (60.1%) patients were prescribed with adjuvant chemotherapy; regimen of TP (taxol and platinum) (73.1%) was the most commonly used regimen in this population. Other chemotherapeutic schemes included TPF (taxol, platinum and 5-fluorouracil) and PF (platinum and 5-fluorouracil). For advanced hypopharyngeal cancer patients with adverse pathologic features, postoperative radiotherapy, either 3D conformal RT or IMRT was performed. The range of total prescribed radiation doses was 60–66 Gy for tumor bed, and 50–56 Gy for lymphatic drainage area (2.0 Gy/fraction; daily Monday–Friday; in 6–7 weeks).

### Follow-Up Evaluation

After the completion of surgery and adjuvant therapy, patients were followed up every 3 months for the first 2 years, semiannually for the subsequent 3 years, and annually thereafter. The observation of follow-up included physical examination, esophagography, chest X-ray, MRI of hypopharynx and neck. Overall survival (OS) was defined as the time interval from the beginning of treatment to death. Disease-free survival (DFS) was the time from the initiation of therapy to recurrence or death of any cause. Locoregional recurrence-free survival (LRFS) was the duration between the beginning of treatment and the first local recurrence or regional lymph node metastasis. Distant metastasis-free survival (DMFS) was defined as the duration from the date treatment began to the time of distant metastasis.

### Statistical Analysis

Data statistical analysis was carried out by employing SPSS 20.0 software. We chose the median level of LDH to separate all the patients into two groups. The correlation between categorical variables and clinical outcomes (DFS and OS) was analyzed by chi-square test. Kaplan–Meier method was used to evaluate survival distribution (DFS and OS), and the log-rank test was performed to calculate the significant differences of survival between groups. Univariate and multivariate Cox proportional hazard regression model was employed to further find out the risk factors for DFS and OS. Patients’ clinical characteristics including age, gender, T stage, N status, tumor differentiation, surgical margin, vascular invasion, and adjuvant chemotherapy were included as covariates in multivariate analysis. Multivariate analysis was carried out by forward stepwise regression procedure. A two-sided P-value ≤0.05 was deemed statistically significant.

## Results

### Baseline Characteristics

The clinical characteristics of 198 eligible patients diagnosed with hypopharyngeal cancer and underwent surgery were retrospectively collected and evaluated. The median follow-up time was 4.63 years (range: 0.58–13.17 years). All of the patients were divided into two groups: low LDH group (≤178 U/L) and high LDH group (>178 U/L). Among these 198 patients, 193 were males and five were females, with a median age of 58 years (range: 36–79 years). Squamous cell carcinoma was the most common histology, and the rest were carcinosarcoma and sarcoma. With regard to histologic grade, the number of well differentiated and poorly differentiated was 163 and 35, respectively. 167 (84.3%) patients had a history of heavy cigarette smoking, and 115 (58.1%) patients were heavy drinkers. According to the 7th edition TNM cancer staging system of AJCC, 185 (93.4%) patients were presented at stages III–IV, 52 (26.3%) patients were diagnosed with T1–2 stage, and 146 (73.7%) patients were diagnosed with T3–4 stage. With regard to lymph node metastasis status, the patient numbers of N0–1 and N2–3 were 75 (37.9%) and 123 (62.1%) respectively. Patients’ clinical and pathological characteristics are presented in **Table 1**.

**Table 1:** Baseline characteristics of patients with pretreatment serum LDH ≤ and >178 U/L.

Variable	Low level of LDH (N = 101)	High level of LDH (N = 97)	*P* value
**Age, year**			0.269
<60	63	53	
≥60	38	44	
**Gender**			0.684
male	98	95	
female	3	2	
T stage*			0.705
T1	8	4	
T2	21	19	
T3	31	31	
T4	41	43	
N status*			0.092
N0-1	44	31	
N2-3	57	66	
TNM stage*			0.100
I	3	1	
II	3	6	
III	24	12	
IV	71	78	
**Tumor differentiation**			0.122
well differentiated	79	84	
poor differentiated	22	13	
**Surgical margin**			0.937
positive	7	7	
negative	94	90	
**Vascular invasion**			0.444
yes	14	10	
no	87	87	
**Adjuvant chemotherapy**			0.931
yes	61	58	
no	40	39	

LDH, lactate dehydrogenase; TNM, tumor-node-metastasis.

*Tumor-node-metastasis staging system proposed by the 7th edition American Joint Committee on Cancer (AJCC).

### Prognostic Role of Pretreatment Serum LDH for the Whole Group

**Figure 1:**
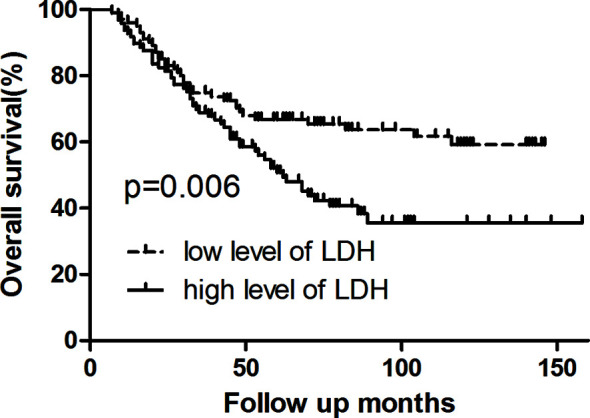
Kaplan–Meier curve for overall survival, stratified by pretreatment serum LDH ≤178 U/L and >178U/L. Log-rank test, P < 0.05.

Survival analysis showed that three- and five-year of DFS (67.0 *vs*. 57.4%, 65.8 *vs*. 39.8%, p = 0.007) and OS (74.8 *vs*. 68.9%, 66.8 *vs*. 50.8%, p = 0.006) exhibited significant difference between low LDH and high LDH groups (**Table 2**). Additionally, three- and five-year of LRFS (low *vs*. high, 73.7 *vs*. 64.9%, 72.6 *vs*. 53.1%, p = 0.024) and DMFS (low *vs*. high, 84.2 *vs*. 79.9%, 81.5 *vs*. 60.6%, p = 0.039) also proved a significant difference between groups (**Table 3**). The Kaplan–Meier curves for OS and DFS for patients with LDH ≤178 U/L and LDH >178 U/L are presented in **Figures 1** **,** **2**.

**Table 2:** Survival analysis for all the patients with pretreatment serum LDH ≤ and > 178 U/L.

Group	Low level of LDH (N = 101)			High level of LDH (N = 97)		P value
	3-year	5-year		3-year	5-year	
DFS	67.0%	65.8%		57.4%	39.8%	0.007
OS	74.8%	66.8%		68.9%	50.8%	0.006

LDH, lactate dehydrogenase; DFS, disease-free survival; OS, overall survival.

**Table 3:** Recurrence Patterns for all the patients with pretreatment serum LDH ≤ and > 178 U/L.

Group	Low level of LDH (N = 101)			High level of LDH (N = 97)		P value
	3-year	5-year		3-year	5-year	
LRFS	73.7%	72.6%		64.9%	53.1%	0.024
DMFS	84.2%	81.5%		79.9%	60.6%	0.039

LDH, lactate dehydrogenase; LRFS, local recurrence-free survival; DMFS, distant metastasis-free survival.

**Figure 2:**
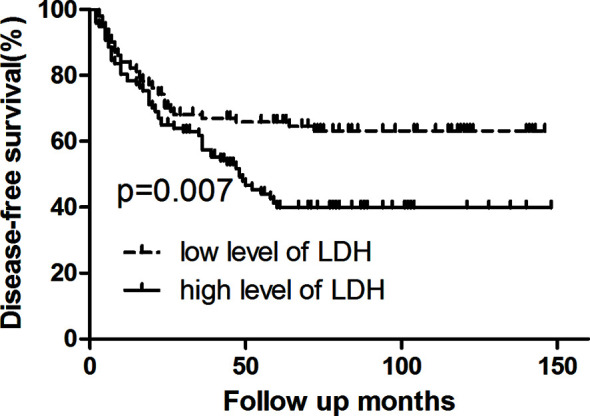
Kaplan–Meier curve for disease-free survival, stratified by pretreatment serum LDH ≤178 U/L and >178U/L. Log-rank test, P < 0.05.

Univariate and multivariate analyses were performed to evaluate the relationship between clinicopathologic variables and survival outcomes. According to univariate analysis, high pretreatment serum LDH served as an unfavorable determinant with regard to DFS (low *vs*. high, p = 0.008; HR = 0.566, 95% CI 0.372–0.863) and OS (low *vs*. high, p = 0.007; HR = 0.560, 95% CI 0.366–0.856). Additionally, age was also proven to be correlated significantly with both DFS and OS, and N status was correlated with DFS. However, gender, T stage, tumor differentiation, surgical margin, and so on were not associated with survival prognosis (**Table 4**). Moreover, multivariate analysis further revealed that LDH (low *vs*. high, DFS, p = 0.031; HR = 0.628, 95% CI 0.410–0.959; OS, p = 0.023; HR = 0.608, 95% CI 0.397–0.933), N status (N0–1 *vs*. N2–3, DFS, p = 0.005; HR = 0.517, 95% CI 0.324 -0.823; OS, p = 0.035; HR = 0.611, 95% CI 0.387–0.967) and age (<60 *vs.* ≥60, DFS, p = 0.007; HR = 0.562, 95% CI 0.369–0.857; OS, p = 0.014; HR = 0.586, 95% CI 0.383–0.897) were independent prognostic factors for both DFS and OS (**Table 5**).

**Table 4:** Univariate analysis of DFS and OS for the Whole Group.

Variable	DFS		OS	
	HR(95%CI)	*P* value	HR(95%CI)	*P* value
**Age, year**				
<60 *vs* ≥60	0.612(0.406–0.925)	0.020	0.623(0.411–0.944)	0.026
**Gender**				
Male *vs* female	3.079(0.429–22.102)	0.263	2.941(0.410–21.113)	0.283
**LDH level**				
Low *vs* high	0.566(0.372–0.863)	0.008	0.560(0.366–0.856)	0.007
**T stage**				
T1–2 *vs* T3–4	1.017(0.638–1.621)	0.945	1.057(0.657–1.700)	0.820
**N status**				
N0–1 *vs* N2–3	0.550(0.349–0.867)	0.010	0.647(0.414–1.011)	0.056
**Tumor differentiation**				
well *vs* poorly	1.076(0.627–1.847)	0.789	0.972(0.566–1.669)	0.919
**Surgical margin**				
Positive *vs* negative	0.973(0.643–1.472)	0.897	0.969(0.641–1.467)	0.883
**Vascular invasion**				
Yes *vs* no	1.328(0.999–1.767)	0.051	1.135(0.827–1.556)	0.433
**Adjuvant chemotherapy**				
Yes *vs* no	1.353(0.882–2.075)	0.166	1.401(0.909–2.159)	0.126

LDH, lactate dehydrogenase; DFS, disease-free survival; OS, overall survival; CI, confidence interval; HR, hazard ratio.

**Table 5:** Multivariate analysis of DFS and OS for all the patients.

Variable	DFS		OS	
	HR(95%CI)	*P* value	HR(95%CI)	*P* value
**LDH** low *vs* high	0.628(0.410–0.959)	0.031	0.608(0.397–0.933)	0.023
**N status** N0–1 *vs* N2–3	0.517(0.324–0.823)	0.005	0.611(0.387–0.967)	0.035
**Age, year** <60 *vs* ≥60	0.562(0.369–0.857)	0.007	0.586(0.383–0.897)	0.014

LDH, lactate dehydrogenase; DFS, disease-free survival; OS, overall survival; CI, confidence interval; HR, hazard ratio.

### Prognostic Role of Pretreatment Serum LDH for T3–4 Patients

Further subgroup analysis exhibited that no significant tendency was observed between LDH and survival outcomes in the T1–2 group (**Table 6**). However, in the T3–4 group, high level of LDH was correlated significantly with worse survival outcomes (low *vs.* high; 3-year DFS: 66.3 *vs*. 55.0%, 5-year DFS: 66.3 *vs*. 40.3%, p = 0.012; 3-year OS: 76.0 *vs*. 68.7%, 5-year OS: 68.5 *vs*. 52.7%, p = 0.008) (**Table 7**).

**Table 6:** Survival analysis for patients with T1–2.

Group	Low level of LDH (N = 29)			High level of LDH (N = 23)		P value
	3-year	5-year		3-year	5-year	
DFS	69.0%	64.4%		65.2%	39.5%	0.345
OS	71.6%	62.0%		69.6%	45.2%	0.397

LDH, lactate dehydrogenase; DFS, disease-free survival; OS, overall survival; CI, confidence interval; HR, hazard ratio.

**Table 7:** Survival analysis for patients with T3–4.

Group	Low level of LDH (N = 72)			High level of LDH (N = 74)		P value
	3-year	5-year		3-year	5-year	
DFS	66.3%	66.3%		55.0%	40.3%	0.012
OS	76.0%	68.5%		68.7%	52.7%	0.008

LDH, lactate dehydrogenase; DFS, disease-free survival; OS, overall survival.

Furthermore, multivariate analysis of DFS and OS for patients with T3–4 showed that LDH was an independent prognostic factor for both DFS (low *vs*. high, p = 0.038; HR = 0.590, 95% CI 0.359–0.971) and OS (low *vs*. high, p = 0.009; HR = 0.516, 95% CI 0.313–0.848), whereas N status (N0–1 *vs*. N2–3, p = 0.012; HR = 0.491, 95% CI 0.282–0.853) and age (<60 *vs*. ≥60, p = 0.008; HR = 0.516, 95% CI 0.316–0.844) were independent prognostic factors for only DFS (**Table 8**).

**Table 8:** Multivariate analysis of DFS and OS for patients with T3–4.

Variable	DFS		OS	
	HR(95%CI)	*P* value	HR(95%CI)	*P* value
**LDH** low *vs* high	0.590(0.359–0.971)	0.038	0.516(0.313–0.848)	0.009
**N status** N0–1 *vs* N2–3	0.491(0.282–0.853)	0.012	NA	
**Age, year** <60 *vs* ≥60	0.516(0.316–0.844)	0.008	NA	

LDH, lactate dehydrogenase; DFS, disease-free survival; OS, overall survival.

## Discussion

Among LDH family members, LDH-5, consisting LDHA subunit only, is the one that has the most vigorous capacity to catalyze pyruvate into lactic acid. Despite the availability of oxygen and the much less ATP produced, cancer cells prevailingly convert into aerobic glycolysis, which contributes to the synthesis of the substrates needed for cancer cell proliferation. Liang S et al. reported that Phosphoenolpyruvate carboxykinase 1 (PCK1) inhibited LDHA expression and thus impeded tumor cells’ growth and metastasis (39). In nasopharyngeal carcinoma, JMJD2A was predicted to boost the Warburg effect through LDHA activation, and fuel tumorigenesis and progression ultimately (40). Jiujie C et al. discovered that FOXM1 and LDHA were overexpressed simultaneously in pancreatic cancer. FOXM1 upregulated LDHA expression by promoting its transcription and was further attributed to pancreatic cancer cell proliferation and metastasis (41). The previous findings of LDH demonstrated the important impact of LDH on tumor development, and therefore, the clinical significance of LDH in cancer merits further investigation.

Elevated serum LDH has long been regarded as an adverse prognostic factor in various malignancies (36). Faloppi L et al. suggested that high pretreatment serum LDH levels significantly associated with poor clinical outcomes in biliary tract cancer patients receiving first-line chemotherapy (30). Pelizzari G et al. reported that during first-line treatment in metastatic breast cancer patients, elevated serum LDH levels served as independent ominous prognostic factor for PFS (33). Additionally, several meta-analysis studies revealed that elevated pretreatment serum LDH was an inferior factor in metastatic prostate cancer (42), osteosarcoma (43), urothelial carcinoma (44), breast cancer (45) and nasopharyngeal carcinoma (46). Moreover, in addition to serving as a prognostic factor, serum LDH also emerges as an indicator of treatment option. Previous studies found that serum LDH was able to distinguish renal cell carcinoma patients who were more likely to benefit from TORC1 inhibition temsirolimus (47), and identify locally advanced cervical cancer patients who could take advantage of neoadjuvant chemotherapy (37). Nevertheless, the reports about LDH’ value in hypopharyngeal cancer is scarcely any.

Hypopharyngeal cancer is a head and neck squamous cell carcinoma, predominantly debuted with advanced stage and accompanied with poor oncologic outcomes. To our best information, our study, for the first time, evaluated the prognostic impact of pretreatment serum LDH in hypopharyngeal cancer patients who underwent primary surgery, and the results confirmed that high level of LDH was an inferior factor in terms of DFS (low *vs*. high, p = 0.008; HR = 0.566, 95% CI 0.372–0.863) and OS (low *vs*. high, p = 0.007; HR = 0.560, 95% CI 0.366–0.856).

More importantly, our results showed that the significant correlation between LDH and survival outcomes was present specifically in T3–4 patients, rather than in T1–2 patients. Additionally, multivariate analysis of DFS and OS for patients with T3–4 showed that LDH was an independent prognostic factor for both DFS (low *vs*. high, p = 0.038; HR = 0.590, 95% CI 0.359–0.971) and OS (low *vs*. high, p = 0.009; HR = 0.516, 95% CI 0.313–0.848). Besides, multivariate analysis also disclosed that N2–3 and age ≥60 were correlated with worse prognosis, and these results were consistent with previous study (15, 48, 49). Based on the findings above, patients with high LDH, N2–3 and age ≥60 tend to experience worse survival outcomes, and this group of patients may need to adopt more intensive follow-up care.

One former study showed that prognostic nutritional index (PNI) played an important role in predicting survival outcomes in hypopharyngeal cancer patients who received surgery; however, the population enrolled in this study was relatively small (48). Recent studies also looked into inflammation index such as NLR and CPR (50, 51). The superior role of NLR, CPR, and LDH in predicting survival was controversial; nevertheless, it may be of great value to incorporate the parameters above in evaluating hypopharyngeal cancer patients prognosis (52, 53).

Nasopharyngeal cancer (NPC) is another head and neck cancer that affects the throat, and the role of LDH in nasopharyngeal cancer had been exhaustively evaluated. Jun M et al. reported that high baseline LDH levels were related with advanced stage and served as an inferior predictor for OS, DFS, and DMFS for NPC patients. Patients with elevated LDH levels may benefit from more aggressive treatment modalities (54). However, Ming-huang H et al. found that LDH was too, a poor predictor for local relapse-free in survival NPC patients (55). For metastatic NPC patients, elevated LDH also was an adverse prognostic factor (56). Moreover, longitudinal variation of serum LDH predicted chemotherapy response in metastatic NPC (57). In addition, prognostic nomogram incorporating LDH and other variables showed satisfactory efficacy in predicting OS of NPC patients (58, 59).

Meanwhile, our study still has certain limitations. First, the present study was performed retrospectively, and therefore, more prospective studies are urgently desired to validate our analysis. Second, the cut-off LDH value was to define the higher LDH level; however, different institutions may have various parameters, which limit the utility of this LDH cut-off value. For patients with advanced disease, concurrent cisplatin-based chemotherapy and radiotherapy is recommended (60), for having equivalent treatment efficacy and maintaining larynx function intact compared to surgery (10–12, 61, 62). However, with radical surgery as the major treatment modality in our institution, the present study failed to investigate LDH prognostic value in patients who received definitive chemoradiotherapy as their initial treatment option. Moreover, our study did not evaluate the expression level of LDHA in tumor samples. Further investigation of the expression levels of LDHA in tumor tissues, its relation with serum LDH, and the clinical significance is warranted. Besides, we noticed significantly low incidence rate (2.5%) of female hypopharyngeal cancer patients during the data collecting process. In contrast, the incidences of female hypopharyngeal cancer in America (16.3–20%) were reported to be higher than that in our study (1, 63). However, our data regarding the low incidence of female patients was supported by other studies carried out in China, Japan, and Korea (1.6–6.2%) (48, 61, 64). Therefore, our results and conclusions may have limited utility for the female population. Above all, our study shed some light on LDH clinical utility in predicting hypopharyngeal cancer patients’ oncological outcomes.

## Conclusions

Pretreatment serum LDH is an independent prognostic factor in hypopharyngeal cancer patients who underwent primary surgery. High pretreatment serum LDH predicts poor prognosis. Patients with high LDH, N2–3 and age ≥60 are inclined to experience worse survival outcomes. Therefore, more intensive follow-up care should be given to these patients.

## Data Availability Statement

The data analyzed in this study is subject to the following licenses/restrictions: institutional data constrained by IRB. Requests to access these datasets should be directed to liuyimincn@139.com.

## Ethics Statement

The studies involving human participants were reviewed and approved by the Institutional Review Board at Sun Yat-Sen Memorial Hospital, Sun Yat-Sen University. Written informed consent for participation was not required for this study in accordance with the national legislation and the institutional requirements.

## Author Contributions

JW was involved in the design, data collection, and writing. YL and XQ were taking part in conception and revision. KY and CC were involved in conception, data analysis, writing, and revision. All remaining authors took part in data collection and writing. All authors contributed to the article and approved the submitted version.

## Conflict of Interest

The authors declare that the research was conducted in the absence of any commercial or financial relationships that could be construed as a potential conflict of interest.
